# ARG2 and NOS2 as predictive biomarkers in renal cell carcinoma

**DOI:** 10.1186/2051-1426-3-S2-P110

**Published:** 2015-11-04

**Authors:** Virginia White, Lucio Miele, John Estrada, Jennifer Cvitanovic, Arnold Zea

**Affiliations:** 1 Dillard's University, New Orleans, LA, USA; 2 LSUHSC-NO, New Orleans, LA, USA; 3 LCRC, New Orleans, LA, USA

## Background

There is a great deal of variability in the survival of patients diagnosed with renal cell carcinoma (RCC) that is not explained by the classical clinic-pathological measures of aggressiveness such as tumor stage or tumor grade. There is an urgent need to identify more accurate molecular and/or metabolic signatures that are predictive of tumor behavior. Such signatures should improve our ability to quantify the risk of progression in individual RCC cases and make accurate prognostic predictions. These measures may also allow oncologists to personalize therapy and identify new targets for drug development. To date, most biomarker research in RCC has centered on von Hippel-Lindau mutations, VEGF, and hypoxia-inducible factors.

## Methods

We have previously shown that increases in the enzyme arginase 2 (ARG2) in RCC tumors is associated with increased polyamine synthesis by tumor cells to sustain their rapid proliferation. In contrast, high levels of nitric oxide synthase (NOS2) and nitric oxide (NO) are associated with slower tumor growth. We sought to validate these findings and determine whether plasma ARG2 is a putative biomarker of RCC activity. analyzed samples obtained from the LCRC Bio-repository.

## Results

We found that ARG2 is expressed at high levels in high-grade clear RCC compared to low-grade RCC. Concomitantly, we observed that NOS2 expression was weakly expressed in high ARG2-expressing tumors. Expression of the two enzymes were confirmed by enzymatic assays, Western blots and HPLC. High ARG2 and low NOS2 expression strongly correlate (r=0.913) with disease progression and short disease-free survival, in contrast to low ARG2 and high NOS2. Importantly, when we tested the levels of ARG2 in the blood of 32 (12 African American and 20 Caucasians) RCC patients, we found significantly higher expression of ARG2 (p=0.02) in African Americans compared to Caucasians and normal controls.

## Conclusions

The results of this study indicate that: 1) ARG2 and NOS2 expression in RCC can be sensitive indicators of tumor activity; 2) Plasma ARG2 may be a useful predictive biomarker and may be linked to health disparities; 3) ARG2 may be a promising therapeutic target in RCC, particularly in African-American patients.

